# Investigating the potential scabicidal activities of three essential oils: gaining mechanistic insights through in vitro contact bioassay and molecular docking

**DOI:** 10.1186/s12906-025-04868-0

**Published:** 2025-05-22

**Authors:** Rofida Wahman, Shaymaa Mohamed, Soad Bayoumi, Rana Morsy, Salma Shafie, Nada Abdelraheem, Fatma Abdelaziz, Salma Hussein, Radwa Ibrahim, Norhan Mohammed, Doaa Yones, Sara Mohammed, Alzahraa Ahmad

**Affiliations:** 1 Department of Pharmacognosy, Faculty of Pharmacy, Assiut University, Assiut, 71526 Egypt; 2 Department of Pharmacognosy and Pharmaceutical Chemistry, College of Pharmacy, Taibah University, Al Madinah Al Munawarah, 41477 Saudi Arabia; 3 Department of Pharmaceutical Organic Chemistry, Faculty of Pharmacy, Assiut University, Assiut, 71526 Egypt; 4 Center for Pharmaceutical Studies and Research for Medicinal Plants, Faculty of Pharmacy (undergraduate student), Assiut University, Assiut, 71526 Egypt; 5 Department of Clinical Pharmacy (Master’s student), Faculty of Pharmacy, Assiut University, Assiut, 71526 Egypt; 6 Department of Medical Biochemistry, Faculty of Medicine, South Valley University, Qena, 83523 Egypt; 7 Department of Parasitology, Faculty of Medicine, Assiut University, Assiut, 71515 Egypt; 8 Department of Veterinary Parasitology, Faculty of Veterinary Medicine, Assiut University, Assiut, 71515 Egypt

**Keywords:** Cassia, Myrrh, Fennel, Scabies, Gas chromatography-mass spectrometry, Docking, In Silico

## Abstract

**Background:**

Scabies, sarcoptic itch, is a highly contagious and pruritic skin inflammation. Given their ecological benefits and previous therapeutic properties, essential oils are worth investigating as potentially safer alternatives to synthetic anti-scabies agents.

**Objectives:**

This study aimed to evaluate the effectiveness of three essential oils derived from cassia barks, myrrh oleo-gum-resin, and fennel fruits against *Sarcoptes scabiei* (Linnaeus, 1758), the causative agent of scabies, using lemongrass and clove essential oils as standards. Additionally, the study explored the structure-activity relationship by characterizing the chemical compositions of these essential oils. Further, a molecular docking study was performed to get further insights into the mechanism of the scabicidal effect of the active essential oils.

**Methods:**

Essential oils were extracted by hydro-distillation from dried cassia, myrrh, and fennel using the Clevenger apparatus, and their chemical profiles were characterized using gas chromatography-mass spectrometry (GC-MS) analysis. The contact bioassay method was utilized to assess their scabicidal activities. The molecular docking study incorporated two target enzymes, glutathione transferase (GST) and inactive serine proteases of scabies mite (SMIPP-S-D1). This is owing to the involvement of these two enzymes in the scabies defense mechanisms.

**Results:**

The GC-MS analysis identified (*E*)-cinnamaldehyde as the primary constituent in cassia essential oil, while β-ocimene, α-copaene, and *trans*-α-bisabolene were major components of myrrh essential oil. Fennel essential oil predominantly consisted of estragole. The contact bioassay demonstrated noteworthy scabicidal activities of cassia and myrrh essential oils, against all tested stages of *S. scabiei*. The docking analysis revealed higher binding affinities between the main phytochemicals of these active essential oils and GST, with binding scores ranging from − 7.7 to − 5.3 kcal/mol, compared to lemongrass and clove essential oils’ main components. Additionally, these components displayed favorable binding affinities to SMIPP-S-D1 ranging from − 5.5 to − 4.0 kcal/mol, comparable to lemongrass and clove essential oils’ main components. These findings suggest that cassia and myrrh essential oils could inhibit the defense mechanisms of scabies mites.

**Conclusion:**

These findings revealed the potential for anti-scabies of essential oils from cassia bark and myrrh oleo-gum-resin, which could effectively control scabies. The Docking analysis showed strong to moderate binding affinities of major components of cassia and myrrh essential oils towards GST and SMIPP-S-D1 enzymes.

**Supplementary Information:**

The online version contains supplementary material available at 10.1186/s12906-025-04868-0.

## Introduction

Plant-derived products have gained recognition for addressing various vector-borne diseases due to their eco-friendliness and biological and medicinal interest. Essential oils (EOs) are plant-derived natural products comprising various terpenes, sesquiterpenes, and aromatic components. They contribute to plant defense mechanisms and possess antibacterial, antiviral, antifungal, and antiparasitic effects. In addition, they aid in patient recovery by offering antioxidant, anti-inflammatory, and immunomodulatory activities [1]. Herbal medications such as essential oils are significant in tropical and subtropical countries, especially Asia and Africa. They have ovicidal, larvicidal, and adulticidal effects against many insects, mite pests, fungi, and bacteria [2, 3].

*Sarcoptes scabiei var. hominis* is an ectoparasite responsible for the emergence of scabies (skin disease) in humans. Scabies infection is a public health problem that affects more than 130 million people globally. In general, scabies cause discomfort due to severe itching. Despite commercially available drugs, pyrethroids and ivermectin, the itching lesions induce significant morbidity due to secondary sepsis and post-infective complications with *Streptococcus pyogenes* and *Staphylococcus aureus* [4].

In Egypt, scabies remain a significant health issue particularly in rural regions, due to a multifaceted set of causes, notably poor living conditions and low parental education levels [5–7]. In 2017 the World Health Organization listed scabies as a neglected tropical disease in isolated areas. In such regions, treating scabies is challenging for economic, environmental, and political reasons. Scabies control is obstructed by diagnosis difficulty, treatment cost, evidence for impending resistance, and effective vaccines. The anti-scabies therapy research has faced considerable challenges in acquiring adequate mite populations. Further, resistance cases to pyrethroids and ivermectin have been increasingly reported. Furthermore, synthetic acaricides have been reported to cause mild to severe adverse effects [8]. Therefore, the necessity for new anti-scabies agents to overcome the disadvantages of current medication was hypothesized [9].

Several essential oils have been investigated, both in vitro and in vivo, against *S. scabiei* from different animal hosts. In some parts of the world, a topical application of 5% tea tree (*Melaleuca alternifolia* (Maiden & Betche) Cheel, family: Myrtaceae) essential oil combined with benzyl benzoate or in the cream formulation has been used to manage scabies with a high cure rate [3, 10]. Additionally, Fang and coauthors, 2016, reported that tea tree, clove (*Syzygium aromaticum*, family: Myrtaceae), palmarosa (*Cymbopogon martini*, family: Poaceae), and eucalyptus (*Eucalyptus globulus*, family: Myrtaceae) essential oils showed potent scabicidal activity. This has the potential to treat scabies infections in humans or animals, besides controlling the mites in the environment. Further, clove, nutmeg, and ylang-ylang essential oils were investigated and showed high, moderate, and weak toxicity against scabies mites, respectively. Their toxicity against mites was due to eugenol and its analogs, acetyl eugenol, and isoeugenol [11].

Studies have shown that neem (*Azadirachta indica* A. Juss., family: Meliaceae) essential oil exhibits acaricidal activity against *S. scabiei*. This effect is attributed to its octadecanoic acid-tetrahydrofuran-3,4-diyl ester content, which damages the body wall of the mites. Additionally, neem essential oil disrupts mitochondrial activity and the oxidative phosphorylation pathway, leading to the parasites’ death. Moreover, an ointment containing 20% aqueous and methanolic neem kernel extracts has been found to have strong acaricidal activity in naturally infected sheep within 20 days of treatment [1]. In addition, lemon essential oil has been reported to possess a strong miticidal activity in both in vitro and in vivo studies. Treatment with 20% lemon essential oil has shown significant improvements in naturally sarcoptic mange-infected rabbits. By the second week, a complete cure was achieved, along with noticeable accelerated hair regrowth [12]. Similarly, the effectiveness of 20% lippia essential oil against scabies mites was found to surpass that of benzyl benzoate. This superior efficacy can be attributed to terpineol, α- and β-pinene activity in lippia essential oil [13]. *Cedrus deodara* essential oil also has shown remarkable effectiveness in controlling sarcoptic mange in sheep [14]. Another study highlighted the significant therapeutic benefits of *Jatropha curcas* essential oil for treating sarcoptic mange of sheep, with ascorbic acid acting as an adjunct therapy, resulting in enhanced meat and wool production [15].

Carvacrol, eugenol, and geraniol have been found to have significant ovicidal activity against *S. scabiei* with low half-maximal effective concentrations (EC_50_) up to 0.5% [16]. Additionally, *Cinnamomum zeylanicum* and *Ocimum sanctum* were the most active against *S. scabiei* among the 31 essential oils tested [8]. Considering that volatile oils have displayed promising potential in combating *S. scabiei* at various stages of its life cycle, it is crucial to assess essential oils containing eugenol, terpineol, α- and β-pinene, or their respective functional groups for their efficacy in treating *S. scabiei*.

Cassia, myrrh, and fennel are aromatic plants with a long history of traditional medicinal use. Several studies have demonstrated the antimicrobial effects of these essential oils against a wide range of bacteria and fungi [17]. Myrrh volatile oil and its crude extracts have shown various pharmacological activities, including antimicrobial, anti-inflammatory, cytotoxic, and sedative actions [18]. Cassia bark extracts have also exhibited wide-ranging anti-inflammatory and antibacterial activities against Gram-negative and Gram-positive bacteria [19]. For example, Cassia bark EO has been found to possess antibacterial effects, with documented anti-staphylococcal activity [20]. Furthermore, it has been tested with classic antibiotics against multidrug-resistant bacteria [21].

Furthermore, Fennel (*Foeniculum vulgare* Mill, Family-Apiaceae) is an essential aromatic plant with well-defined medicinal properties, including anti-inflammatory and antimicrobial activities [22]. Notably, fennel essential oil has shown acaricidal and repellent effects against *Tetranychus urticae* mites [23].

Although these plants are known for their antimicrobial, anti-inflammatory, and antioxidant properties, their potential applications in scabies treatment have not been investigated. The antioxidant properties of these essential oils may help protect cells from damage caused by oxidative stress, which is implicated in the pathogenesis of scabies. Moreover, their anti-inflammatory effects may aid in reducing the inflammation associated with scabies, thereby addressing discomfort and preventing secondary infections. Therefore, their valuable biological properties suggest a potential therapeutic value for scabies treatment, although further investigation is needed to confirm their efficacy.

To the authors’ knowledge, this is the first study to evaluate the scabicidal activities of cassia, myrrh, and fennel essential oils against *S. scabiei* mites. Additionally, this study involves characterizing the active main components present in these essential oils. Moreover, the investigation includes valuable mechanistic insights revealed through molecular docking studies.

## Materials and methods

### Plant materials, chemicals, and reagents

The herbal materials, include cassia barks (*Cinnamomum cassia*, Family: Lauraceae), myrrh oleo-gum-resin (*Commiphora myrrha*, Family: Burseraceae), fennel fruits (*Foeniculum vulgare*, Family: Apiaceae), and clove flower buds (*Syzygium aromaticum*, Family: Myrtaceae), were purchased from a local market at Assiut City, Egypt. Lemongrass essential oil (100% purity, artnaturals^®^, Woodland Hills, CA, USA) was used. Paraffin oil was obtained from Pure Misr Company, Egypt.

### Preparation of essential oils

Cassia, myrrh, fennel, and clove were milled to fine powder. They were extracted using a hydrodistillation method using a Clevenger apparatus for 4 h at normal pressure. The essential oils were collected and kept in the fridge until GC-MS analysis.

### Gas-chromatography-mass spectrometry (GC-MS)

The collected essential oils were analyzed to identify their constituents using Agilent GC-MS (7890-5975MSD). The run was 48 min, with two posts each running 51 min. The chromatographic system consists of a DB-5MS capillary column (30 m × 0.25 mm × 0.25 μm) with helium as a carrier gas. The experiment was performed with the following conditions: a maximum inlet temperature of 280 °C. It started at 40 °C for 2 min, then was increased to 150 °C for 6 min. The temperature increased to 220 °C for 6 min and 280 °C for 15 min with the rate of 10 °C/min except for the last one with 15 °C/min. The flow rate was programmed: 0.5 mL/min for 10 min then 1 mL/min for 30 min. The peaks were detected with 5975 Quadruple-MS, which ionized with the electron impact at 230 °C, reaching a maximum of 250 °C.

### Experimental animals

This study used ten naturally *Sarcoptes*-infested New Zealand rabbits (Mean weight 1200 ± 300 g) obtained from Assiut University Animal Farm. The infection was confirmed by skin scraping and examination under a stereomicroscope. The infested rabbits were housed at the Faculty of Medicine’s animal care facility and fed a standard diet with clean water. All rabbits were not treated with any miticidal drug before the collection of mites. Sampling and all experimental practices were conducted according to the institutional ethical and animal care guidelines [24]. The study was carried out in compliance with the ARRIVE guidelines [25].

### Collection of *S. scabiei* mites

Crusts of skin lesions from the ears and the back of infested rabbits were gently removed and placed in a Petri dish containing normal saline. Animal sedation before skin scraping was ensured by xylazine administration (0.6 ml I.M.) [26]. The rabbits were not euthanized at the end of the experiment, as no further in vivo procedures were required. Only living motile mites of adults, nymphs, and larval stages were picked up with a fine needle and placed in the middle of a new plastic Petri dish (3 cm in diameter), then incubated immediately at 35 °C for 30 min before the start of experiments. The collected mites were immediately tested under a stereomicroscope for contact bioassay.

### Contact bioassay

The in vitro acaricidal activities of three EOs, cassia, myrrh, and fennel, against *S. scabiei* were evaluated by uniformly impregnating motile mite stages with EOs diluted with paraffin oil at concentrations ranging from 1 to 10%. The collected mites (*n* = 20) were placed directly in contact with 100 µl of each diluted essential oil for each experiment. Negative control mites were treated with paraffin oil, while clove 5% and lemongrass 10% essential oils were used as positive controls as previously described [8, 27]. All experiments were done in triplicate. The survival assessment of mite viability was assessed by stereomicroscopic observation at 30, 60, 90, 120, and 180 min post-treatment. Mites that remained motionless for 5 min and showed no response to needle stimulation were considered dead.

### Scanning electron microscopy

After the experiment, the dead mites were collected and preserved in 2.5% buffered glutaraldehyde at 4 °C overnight. The tubes containing the specimens were centrifuged for 5 min at 1500 rpm. Then, the supernatant was discarded, and the samples were fixed in 1% osmium tetroxide at 4 °C for 1 h. To prepare the specimens for observation, dehydration was performed using ascending grades of acetone (30–100%). The specimens were then dried, mounted on stubs, and coated with gold using standard protocol [28]. The samples were ultimately examined at the Unit of Scanning Electron Microscope at Assiut University using a Zeiss DSM 940 electron microscope.

### Statistical analysis

Survival curves for scabies mites exposed to essential oils were derived using the Kaplan-Meier method. Median lethal times (LT50) were calculated using IBM SPSS software (version 28.0), with censored data for mites that survived beyond 180 min. The Log-rank test evaluated statistical differences between results obtained with each essential oil (at 10%, 5%, and 1% concentrations) and the negative control for each experiment. Results were expressed using chi-square values (χ2) and *p*-values, with degrees of freedom (Df) set to 1. The *p*-value of ≤ 0.05 was considered statistically significant.

### Molecular Docking study

#### Preparation of modeled proteins

The X-ray crystallographic structures of protein targets (GST (PDB 3EIN), resolution 1.13 Å, GSH as the co-crystallized ligand) [29] and protease (PDB 3H7T, resolution 2.00 Å [30] ) were obtained from the Protein Data Bank website (http://www.rcsb.org/pdb/). Docking simulation studies were performed using Autodock vina (version 1.1.2) software as a free and open-source docking tool [31]. For protein preparation, water and uninvolved ligands were removed. Polar hydrogens were added, while nonpolar hydrogens were merged. Gasteiger charges were added, and missing atoms were repaired using Autodock tools (version 1.5.7) [32] for 3EIN and using Chimera X (version 1.7.1) [33] for 3H7T (only the first chain was kept). Atom types were assigned. The protein structures were saved in pdbqt file format.

#### Ligand Preparation

The phytochemical composition of lemongrass essential oil was obtained from relevant literature [27]. The 2D structures of the top 4 most prominent components (ligands) of lemongrass, clove, cassia, and myrrh EOs were retrieved from the PubChem website (https://pubchem.ncbi.nlm.nih.gov/) in sdf format. Ligands were prepared Using Autodock tools (version 1.5.7). Nonpolar hydrogens were merged while polar hydrogens were added. Rotatable bonds were defined, and torsion trees were built for each ligand. Prepared ligand structures were saved in pdbqt file format.

#### Molecular Docking analysis

The binding site on the target proteins, delta class glutathione transferase (GST, PDB 3EIN) and scabies mite inactivated protease paralogue S-D1 (SMIPP-S-D1, PDB 3H7T), were specified by establishing a grid box with a grid spacing of 0.5 Å using Autodock tools (version 1.5.7) [32]. For GST, the grid box was centered on coordinates X: −11.288 Å, Y: 50.721 Å, and Z: 0.564 Å with grid dimensions (grid size) 48, 50, and 40, respectively. For the SMIPP-S-D1, it was centered on coordinates X: 24.385 Å, Y: 83.239 Å, and Z: 24.326 Å with grid dimensions of 50, 28, and 28, respectively. All docking simulations were performed using Autodock vina (version 1.1.2) software [31]. To perform docking, commands were given to Autodock vina (version 1.1.2) software [31] through the command prompt. The exhaustiveness parameter was set to sixty-four for all docking runs. To ensure consistency of the results, one hundred runs were performed for each ligand structure in all cases. The binding affinity (scores) between the ligand and protein was calculated using the search algorithm within the AutoDock Vina software [31]. After the docking runs were completed, binding modes and binding affinity of each protein-ligand interaction were examined in 2D and 3D styles using BIOVIA Discovery Studio visualizer 2024 [34]. Favorable poses that showed the most stable conformation with the lowest binding score and best ligand–enzyme interactions were selected.

#### Docking protocol validation

For the GST model, the vicinity of the GSH (glutathione) ligand was considered the active site (site-specific docking). It was examined by using the Protein Plus website (https://proteins.plus/). It revealed several valuable key hydrogen bonding interactions with (Ser 10, His 51, Ser 66, and Ile 53) and ionic interactions with (Glu 65 and Arg 67) amino acid residues. GSH was re-docked using Autodock vina (version 1.1.2) software for validation (Table S1) and showed comparable interactions. For the protease model, the vicinity where Leu 31, Lys 103, Lys 104 and Lys 225 amino acids are present, was chosen as the active site (site-specific docking). This was based on the binding and mutagenesis studies reviewed from the literature [35].

## Results and discussion

### The major constituents of essential oils (EOs)

The gas chromatography-mass spectrometric analysis of essential oils under study has elucidated the chemical basis of their efficacy. This analysis identified the major constituents of the studied EOs. In addition, our analysis uncovered compounds analogous to the reported components of the reference standards lemongrass and clove EOs, including eugenol, terpineol, as well as α- and β-pinene. Furthermore, it also revealed the presence of other functional groups that may be associated with the activity of essential oils against *Sarcoptes scabiei* mites.

**Fig. 1:**
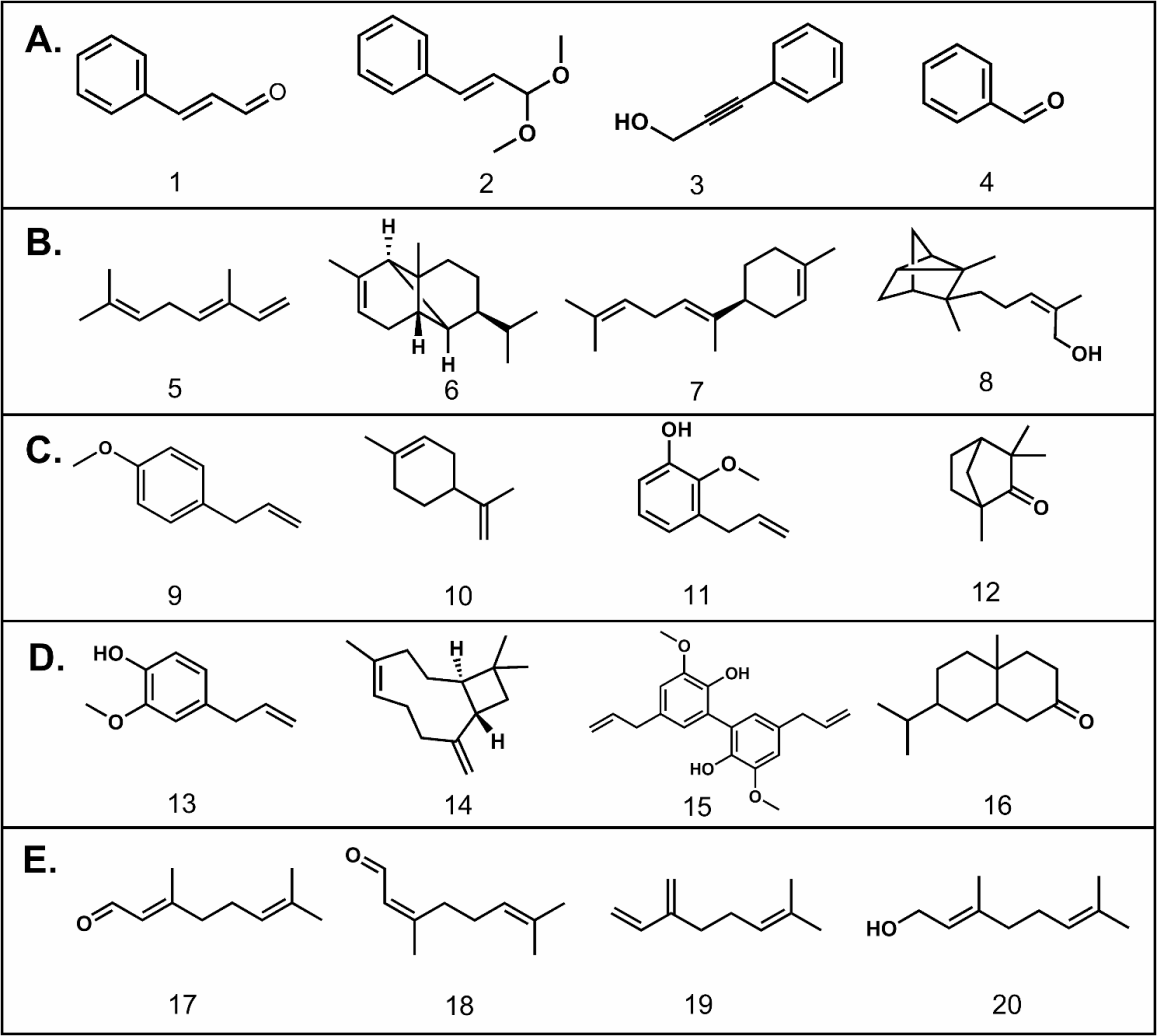
The chemical structures of major constituents of (**A**). Cassia essential oil: (*E*)-cinnamaldehyde (1), cinnamaldehyde-dimethyl acetal (2), 3-phenyl-2-propyn-1-ol (3), benzaldehyde (4); (**B**). Myrrh essential oil: β-ocimene (5), α-copaene (6), *trans*-α-bisabolene (7), α-santalol (8); (**C**). Fennel essential oil: estragole (9), limonene (10), 3-allyl-2-methoxyphenol (11), fenchone (12); (**D**). Clove essential oil: eugenol (13), caryophyllene (14), dehydrodieugenol B (15), 7-isopropyl-4a-methyloctahydro-2(1 H)-naphthalenone (16); and (**E**). Lemongrass essential oil: geranial (17), neral (18), myrcene (19), and geraniol (20)

Regarding the standards, clove, and lemongrass essential oils, the chemical composition of lemongrass essential oil has been reported to contain key compounds including geranial, neral, myrcene, and geraniol. Li and coauthors revealed that the acaricidal activity of lemongrass essential oil stems from a notable proportion of two aldehydes, geranial, and neral in addition to myrcene [27]. Furthermore, clove essential oil chromatogram showed high concentrations of eugenol, dehydrodieugenol B in addition to caryophyllene which collectively participate in the renowned activity of clove against *Sarcoptes scabiei* mites (Table 1; Fig. 1).

**Table 1:** Major constituents of clove flower bud, cassia bark, myrrh oleo-gum-resin, and fennel fruit essential oils with corresponding percentages and retention times

Compound Name	Percentage (%)	RT (min)
**Clove flower buds**		
Eugenol	54.19	15.259
Caryophyllene	23.74	16.618
Dehydrodieugenol B	10.36	19.205
7-Isopropyl-4α-methyloctahydro-2(1 H)-naphthalenone	3.52	17.368
(-)-α-copaene	2.38	15.589
(-)-β-Caryophyllene epoxide	2.22	20.997
(-)-6-α-Cadina-4,9-diene	0.23	17.782
α-Gurjunene	0.33	18.487
Humulene-1,2-epoxide	0.22	21.579
ɣ-Cadinene	0.36	17.847
Chavicol (4-allylphenol)	0.42	13.487
Caryophyllene oxide	0.31	20.926
Total identified components	98.28	
**Cassia bark**		
(*E*)-Cinnamaldehyde	76.80	13.881
Cinnamaldehyde-dimethyl acetal	4.59	15.861
3-Phenyl-2-propyn-1-ol	3.32	13.196
Benzaldehyde	2.63	9.612
*trans*-Cinnamic acid	1.99	16.320
2 H-Chromen-2-one	1.83	16.909
4-Isopropyl-1,6-dimethyl-1,2,3,4-tetrahydronaphthalene	1.53	19.257
*o*-Methoxycinnamaldehyde	1.42	19.354
1,6-Dimethyl-4-(1-methyl ethyl)-naphthalene	0.97	22.853
Benzoic acid	0.77	12.471
Cinnamyl acetate	0.77	16.812
α-Cadinol	0.77	22.265
Total identified components	97.39	
**Myrrh**		
β-Ocimene	22.60	12.392
α-Copaene	14.67	23.467
*trans* -α-Bisabolene	12.09	20.215
α-Santalol	10.95	18.578
6-α-Cadina-4,9-diene	6.03	20.661
*trans*-α-Bergamotene	4.86	18.780
2-Oxo-3-pyrazin-2-yl-propionic acid, ethyl ester	3.58	20.874
β-Bisabolene	3.12	20.374
*(Z*, *E)-*α-Farnesene	2.28	18.440
*Τ*-Cadinol	1.98	22.883
Camphene	1.78	23.892
Total identified components	83.94	
**Fennel fruit**		
Estragole	57.14	12.976
Limonene	13.52	10.803
3-Allyl-2-methoxy phenol	9.80	15.182
Fenchone	7.84	11.754
Anethole	3.54	14.037
ɣ-Terpinene	0.49	11.268
Fenchyl acetate	0.31	13.422
Myrcene	0.33	10.065
Camphor	0.30	12.420
Caryophyllene	1.17	16.547
*E*-Limonene-1,2-epoxide	0.28	12.278
(+)-Carvone	0.63	13.533
Neo-allo-ocimene	0.45	12.129
Total identified components	95.80	

In the cassia bark essential oil, (*E*)-cinnamaldehyde, cinnamaldehyde-dimethyl acetal, 3-phenyl-2-propyn-1-ol, and benzaldehyde were detected as the predominant compounds, constituting the highest concentrations (76.80%, 4.59%, 3.32%, and 2.63%) at retention times of 13.881, 15.861, 13.196, and 9.612 min, respectively. These compounds hold a resemblance to eugenol, renowned for its efficacy against *Sarcoptes scabiei*. Both cinnamaldehyde derivatives feature phenolic and aliphatic chains with double bonds, while the acetal one includes a methoxy group on the aliphatic chain. Additionally, 3-phenyl-2-propyn-1-ol also contains a phenolic moiety. These distinctive functional groups likely contribute to the activity of cassia EO against *Sarcoptes scabiei* mites (Table 1; Fig. 1).

The GC-MS analysis of myrrh EO detected β-ocimene, α-copaene, *trans*-α-bisabolene, and α-santalol at considerable concentrations. β-ocimene shares structural similarities with myrcene due to its unsaturated aliphatic chain. However, α-copaene resembles the structure of caryophyllene. Moreover, *trans*-α-bisabolene and α-santalol have structures resembling pinene (Table 1; Fig. 1).

The GC-MS analysis revealed that fennel EO comprises estragole (57.14%), limonene (13.52%), 3-allyl-2-methoxyphenol (9.8%), and fenchone (7.84%). The 3-allyl-2-methoxyphenol derivative is recognized as a positional isomer to eugenol (4-allyl-2-methoxyphenol). Further, limonene and fenchone share structural similarities to terpineol and pinene (Table 1; Fig. 1). Notably, the positional isomer to estragole (anethole) was present at a limited percentage of 3.54% [12]. This could potentially impact the anticipated efficacy of fennel essential oil.

### The effect of tested essential oils on *Sarcoptes scabiei* mites

**Fig. 2:**
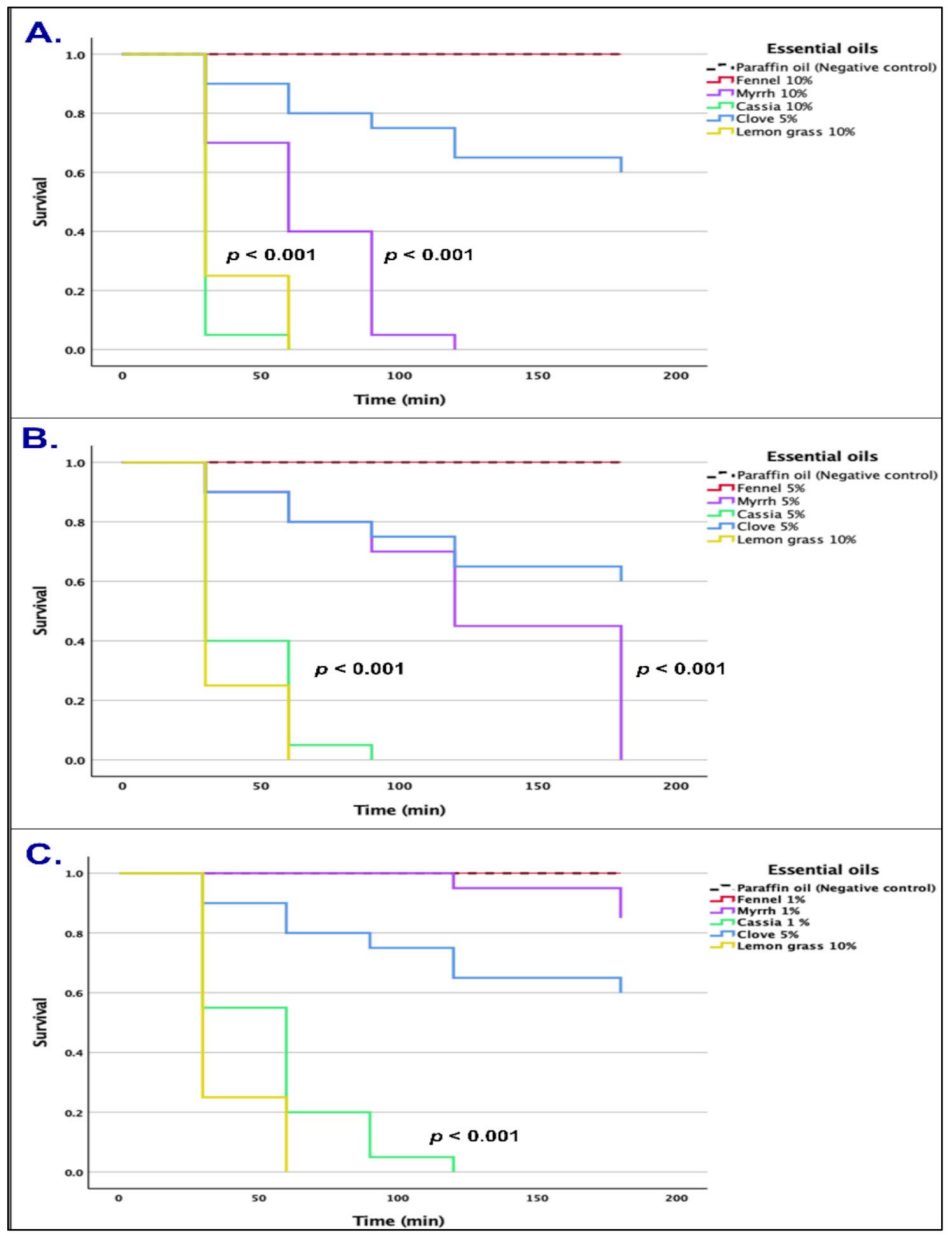
Survival curves of *Sarcoptes scabiei* mites exposed to fennel, myrrh, and cassia essential oils in contact tests at varying concentrations: (**A**). 10%, (**B**). 5%, and **C**. 1%. These are compared against the negative control (paraffin oil) and positive controls (5% clove essential oil and 10% lemongrass essential oil)

Analysis of survival curves of mites exposed to EOs via direct contact at 10%, 5%, and 1% concentrations are depicted in (Fig. 2). Our results demonstrate the potent anti-scabies activity of cassia essential oil, which proved to be the most effective across all EOs tested, resulting in nearly complete mite mortality within 1.5 h of direct contact at 10% and 5% concentrations, and within 2 h at 1% concentration (Table 2). The myrrh essential oil also resulted in the death of almost all mites within 2 h of direct exposure at 10% concentration and within 3 h of exposure at 5% concentration (Table 2). Median lethal times (LT_50_) of the EOs examined at 10%, 5%, and 1% concentrations are presented in (Table 3). Statistical analysis revealed significant differences between cassia essential oil and paraffin oil control across all concentrations (*P* < 0.001). Likewise, myrrh essential oil exhibited substantial differences at concentrations of 10% and 5% (*P* < 0.001), but no difference was observed at 1% compared to the paraffin oil control (*P* = 0.075).

**Table 2:** The mortality rate of scabies mites after exposure to different concentrations of cassia bark, myrrh oleo-gum-resin, and fennel fruit essential oils at different time intervals

Concentration	Mortality rate (± SD), %				
	30 min	60 min	90 min	120 min	180 min
**Cassia essential oil**					
10%	93.3 (0.05)	97.7 (3.15)	100	100	100
5%	59.9 (0.05)	93.3 (0.09)	100	100	100
1%	46.6 0.11	82.1 (0.09)	95.5 (0.06)	100	100
**Myrrh essential oil**					
10%	28.8 (0.03)	59.9 (0.14)	94.4 (0.08)	100	100
5%	11.1 (0.16)	18.3 (0.11)	31.6 (0.11)	53.9 (0.4)	100
1%	0	0	2.2 (0.03)	4.4 (0.03)	15.5 (0.03)
**Fennel essential oil**					
10%	0	0	0	0	0
**Paraffin oil (negative control)**					
	0	0	0	0	0

**Table 3:** Median lethal times (LT50) of the tested essential oils in contact bioassays in comparison with positive controls (in minutes)

Essential oil	Contact with a 10% solution				Contact with a 5% solution				Contact with a 1% solution			
	LT50 ± SE	95% CI	χ2	*P*	LT50 ± SE	95% CI	χ2	*P*	LT50 ± SE	95% CI	χ2	*P*
Lemongrass	30*	–	43.071	< 0.001								
Clove					–	–	9.819	0.002				
Cassia	30*	–	41.971	< 0.001	30*	–	43.156	< 0.001	60 ± 7.667	[44.974 − 75.026]	44.466	< 0.001
Myrrh	60 ± 10.954	[38.529 − 81.471]	42.138	< 0.001	120 ± 13.349	[93.836 − 146.164]	38.945	< 0.001	–	–	3.160	0.075
Fennel	–	–	–	–	–	–	–	–	–	–	–	–

*Majority of mites died before 30 min

Previous studies have consistently found that cassia essential oil or its constituents possess acaricidal activity. For instance, cassia essential oil has been shown to exhibit fumigant toxicity against *Dermanyssus gallinaewas*, the poultry red mite [36]. Furthermore, two major constituents of cassia essential oil, namely *trans*-cinnamaldehyde, and salicylaldehyde, have demonstrated significant toxicity against house dust mites, particularly *Dermatophagoides farinae* and *Dermatophagoides pteronyssinus* [37]. Additionally, the acaricidal effect of *Cinnamomum cassia* components against *Haemaphysalis longicornis* has also been reported [38]. Interestingly, this is the first report concerning the anti-scabies activity of *Cinnamomum cassia* essential oil.

Our study found that cassia essential oil has a significant scabicidal effect against all stages of *S. scabiei*, in a concentration-dependent manner. Mites were killed within 1.5 h at 10% and 5% concentrations of cassia essential oil and within 2 h at 1% concentration. These results are similar to the scabicidal effects of tea tree essential oil reported by Fang and colleagues. They observed that 10% and 5% concentrations of tea tree essential oil killed *S. scabiei* within 30 and 90 min, respectively [8]. Also, these results demonstrated the potent efficacy of cassia essential oil in comparison with the previously reported potent miticidal essential oils such as lemongrass (*Cymbopogon citronella*) and clove (*Eugenia caryophyllata*) essential oil [27]. Our observations suggest that cassia essential oil is comparable to lemongrass and tea tree essential oils and may even be more effective than clove essential oil when used as a scabicide.

Myrrh is an oleo-gum-resin exudation obtained from the bark of various trees within the *Commiphora* genus. It contains a significant amount of gum (up to 60%), resins, and essential oils [17]. In a previous laboratory study, myrrh showed a potential toxic activity on adult female fowl ticks (*Argas persicus*). The toxic effects have gradually increased daily after the treatment [39]. Consequently, it is essential to conduct additional in vivo studies of cassia and myrrh essential oils to confirm their therapeutic efficacy as scabicides.

Contrary to expectations, the tested concentrations of fennel EO did not exhibit acaricidal activity against *Sarcoptes scabiei*. These findings contradict an earlier study that evaluated the toxicity of seven plant essential oils, including fennel, against *Tetranychus urticae* and two other mite species, *Phytoseiulus persimilis* and *Neoseiulus californicus*. In addition, the acaricidal activity of fennel essential oil was tested previously, and the results of such studies showed its high toxicity against female specimens of the predatory mite *P. persimilis* [40]. It is crucial to note that variations in the composition and concentration of constituents found in EOs can occur within a specific range. This may explain the discrepancies between the current findings and earlier in vitro investigations utilizing fennel essential oil. These differences may result from various environmental factors that impact the plant, including soil type, climate, location, and even fertilizer used [41, 42]. Also, the type of tested parasite and the experimental conditions, including the extraction method of essential oil, could influence the reproducibility of the tested essential oils from one study to another.

Paraffin oil is an effective solvent for in vitro evaluation of essential oils. In the present study, the mites exposed to paraffin oil had the longest mean survival time. A previous study reported that all *Sarcoptes* mites survived after being immersed in paraffin oil for 24 h [27]. Another study tested three different solvents (acetone, paraffin oil, and 20% glycerol) against ear mites (*Otodectes cynotis*). The results showed that paraffin oil was the best solvent due to its properties of solubility, viscosity, and volatility [43].

### Morphological changes of mites using scanning electron microscopy (SEM)

**Fig. 3:**
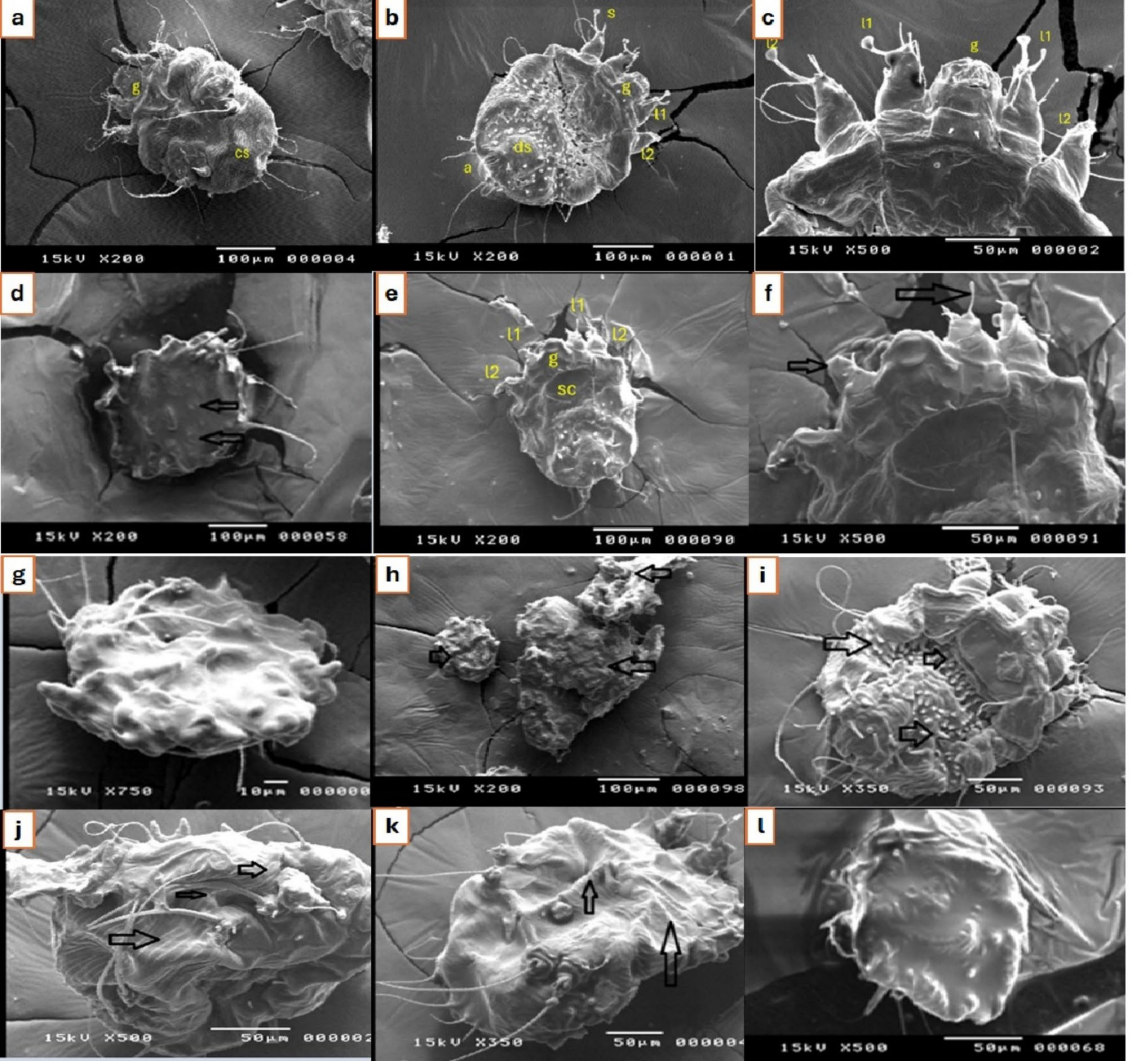
Scanning electron micrographs showing effects of different essential oils on *Sarcoptes scabiei* adult and nymph stages after 24 h of exposure: a–c: normal adult *Sarcoptes scabiei* female in paraffin oil (negative control) showing the normal structures in ventral (**a**) and dorsal views (**b**, **c**). g: gnathostoma; cs: cuticular striations; l1,2: legs; ds: dorsal spines; a: anus; s: suckers. (**d**–**f**) nymph stages of *S. scabiei* (dorsal view) after being treated with myrrh 5%. (**d**) posterior spines are reduced and lost in many parts (arrows). (**e**): gnathostoma (**g**) scutum (sc) showing marked smoothening of the surfaces of the mites; legs (l1,2). (f): closer view to the anterior 2 pairs of legs showing loss of the suckers (arrows). (**g**–*i*) different stages of *S. scabiei* after being treated with cassia 5% (**g** and **h**): cannot be distinguished clearly due to loss of cuticular thumb-like markings, reduced dorsal spines, and marked smoothening of the surfaces of the mites. (**h**): shows an aggregation of three mites (arrows) with observed shrinkage of the mites. (**i**): adult stage of *S. scabiei* (dorsal view) after treatment with cassia 1% showed marked shrinkage of the mite indicating mites’ dehydration with the accentuated appearance of the characteristic dorsal spines (arrows). J–l: adult stage of *S. scabiei* (positive controls) after being treated with clove essential oil 5% (**j** and **k**) (ventral view) showing shrinkage of the mite while visible cuticular striations with thumb-like appearance (arrows) indicating mite’s dehydration, (**l**): *Sarcoptes scabiei* (cannot distinguish the stage or the surface clearly due to marked deformity) treated with lemongrass essential oil 10%. Shrinkage, diminished cuticular marking loss of some legs, and deformity of the mites were observed

The scabicidal effects of the tested essential oils were supported by scanning electron microscope observations that clearly showed the marked deformity, shrinkage, dehydration, and loss of normal structure as dorsal spines and cuticular striations following exposure to cassia and myrrh EOs 5%, compared to positive control essential oils, lemongrass and clove essential oils (Fig. 3). The cassia and clove essential oils have similar effects as they induce dehydration and shrinkage of the mites. This might be due to the similarity in the structure of the phytochemicals between them, especially eugenol and 3-phenyl-2-propyn-1-ol. Moreover, in myrrh, compounds containing substituted benzene ring or unsaturated aliphatic chains are similar in structure to lemongrass and clove essential oil components which might be the active functional groups in inducing the scabicidal effect.

### Molecular Docking studies

Molecular docking studies were performed to rationalize the in vitro biological results and to investigate the mechanism of the scabicidal effect of cassia and myrrh essential oils. The study was performed on cassia and myrrh essential oils’ top four main components and compared to lemongrass and clove essential oils’ main phytochemicals. Notably, this will determine if these compounds could affect the lifecycle of scabies mites and cause their death. All these components were screened against two target proteins extensively involved in the infection process. The selected target GSH transferase enzyme (PDB 3EIN) co-crystalized with glutathione helps detoxify endogenous compounds and xenobiotics in mites. Consequently, this enzyme increases mite resistance to scabicidal agents [44]. The other selected protein was scabies mite inactivated protease paralogue (SMIPP-S-D1) [45]. SMIPP-S-D1 has a chymotrypsin-like serine protease fold but does not perform protease activity. Previous studies have shown that certain conserved amino acid residues blocked the presumed chymotrypsin-like catalytic domain of this enzyme. Notably, it was discovered that SMIPP-S-D1 is a complement pathways inhibitor and a potent lectin pathway inhibitor. Inspection of several scabies’ mite proteases has revealed a conserved lysine residue on the other side of the presumed catalytic domain. This conserved area has included Leu 31, Lys 103, Lys 104, and Lys 225 amino acids which was confirmed by mutagenesis and binding studies. Therefore, the vicinity where these amino acids are located will be used as the active site. It is noteworthy to mention that our docking study on scabies mite (SMIPP-S-D1) will be among the first studies to be performed on this enzyme [45].

**Fig. 4:**
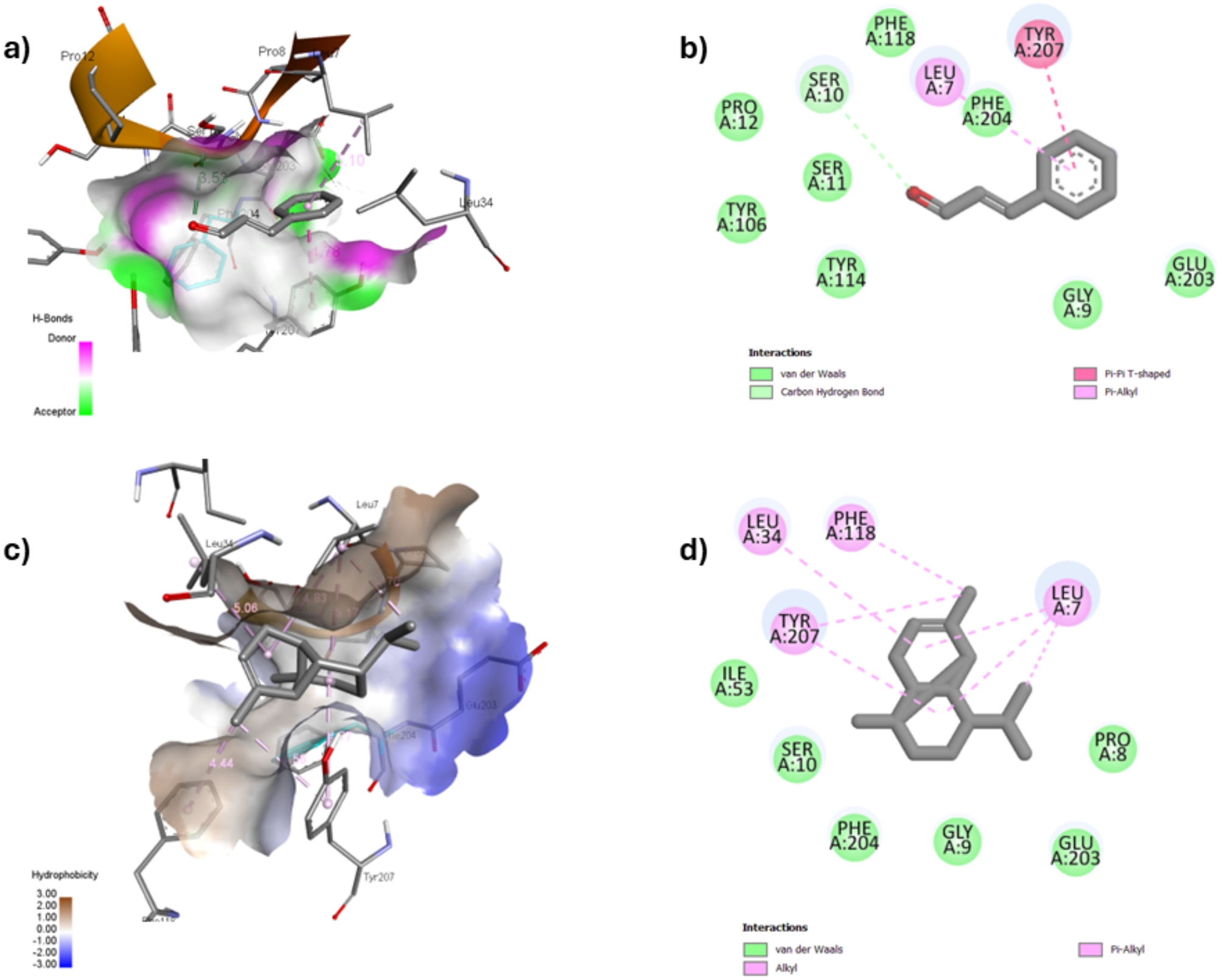
(**a**) 3D interaction, (**b**) 2D interaction of (*E*)-cinnamaldehyde from cassia essential oil, (**c**) 3D interaction, and (**d**) 2D interaction of α-copaene from myrrh essential oil into the active site of GST (PDB 3EIN)

The docking simulation program Autodock vina (version 1.1.2) software was used for computational studies. BIOVIA Discovery Studio Visualizer 2024 [34] was used to visualize the different docked poses. The docking results of the main compounds against GST enzyme (PDB 3EIN) are listed (Table S1). Re-docking of co-crystallized ligand glutathione has shown a binding affinity of − 5.4 kcal/mol (Table S1). The pattern of interactions included amino acids (Thr 53, Ile 53, Glu 65, and Arg 67) as hydrogen bond donors and Ile 53 amino acid as hydrogen bond acceptor. Remarkably, the docking results of cassia and myrrh essential oils’ top four main components showed better affinities toward GST than the co-crystalized ligand except for benzaldehyde (Table S1). These results were comparable to the docking results of lemongrass and clove essential oils’ components (Table S1). (*E*)-Cinnamaldehyde, cinnamaldehyde-dimethyl acetal, and 3-phenyl-2-propyn-1-ol exhibited binding scores (− 5.8, − 6, and − 6.1 kcal/mol, respectively) higher than GSH and interacted with Ser 10 (Carbon H-donor) and Leu 7 (Pi-alkyl) amino acid residues (Fig. 4). In addition, the cinnamaldehyde derivatives showed another hydrophobic interaction with Tyr 207 (pi-pi T-shaped), while the propyne derivative showed the same hydrophobic interaction but with Gly 9.

Notably, myrrh essential oil main components (β-ocimene, α-copaene, *trans*-α-bisabolene, and α-santalol) displayed extensive pi-alkyl and alkyl hydrophobic interactions within the active site of GST. These components exhibited high binding energies ranging from − 7.7 to − 6.0 kcal/mol. These interactions incorporated (Leu 7, Pro 8, Pro 12, Leu 34, Tyr 114, Phe 118, Phe 204, and Tyr 207) as the chief interacting amino acids (Fig. 4).

In addition, the docked components of lemongrass displayed comparable binding scores (ranging from − 6.0 to − 5.6 kcal/mol) to those of the cassia essential oil components (ranging from − 6.1 to − 5.3 kcal/mol). Geranial showed two hydrogen bonding interactions, with Ser 11 and Tyr 106 as the H-donors, along with pi-alkyl and alkyl hydrophobic interactions involving Leu 7, Pro 8, Tyr 207, and Phe 204. Similarly, myrcene demonstrated hydrophobic interactions similar to those observed with geranial.

The di-eugenol derivative and eugenol from clove essential oil displayed two hydrogen bond interactions with Ser 11 and Tyr 114. Notably, di-eugenol showed superior binding affinity (− 7.3 kcal/mol). Both compounds exhibited strong hydrophobic interactions (pi-alkyl and alkyl), especially with Pro 12 and Tyr 207 amino acid residues. Remarkably, the naphthalenone derivative displayed the highest binding affinity (− 7.7 kcal/mol), establishing three strong hydrogen bonds with Ser 11, Tyr 114, and Pro 12 as the H-donors.

**Fig. 5:**
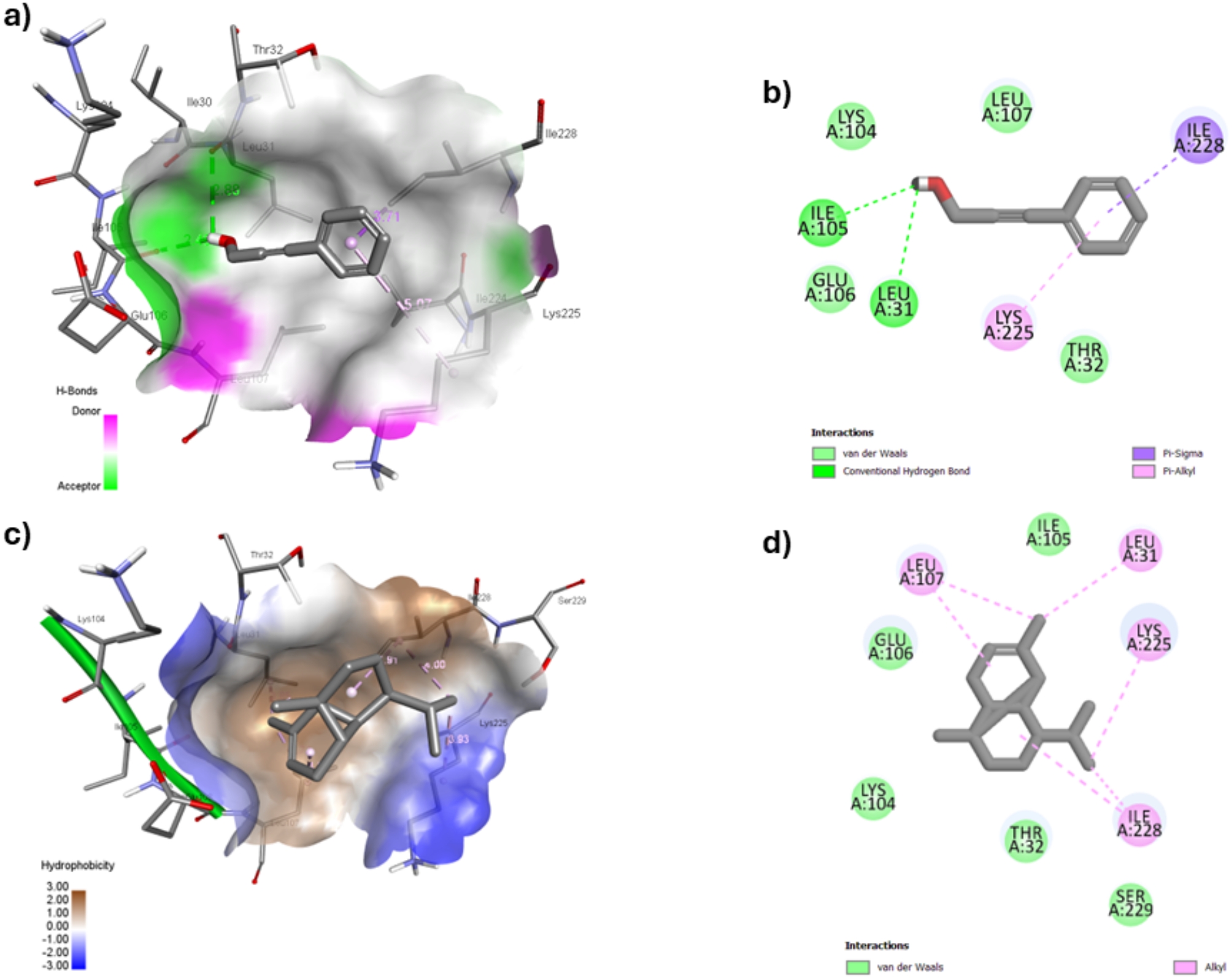
(**a**) 3D interaction, (**b**) 2D interaction of 3-phenyl-2-propyn-1-ol from cassia essential oil, (**c**) 3D interaction, and (**d**) 2D interaction of α-copaene from myrrh essential oil into the active site of SMIPP-S-D1 (PDB 3H7T)

Table S2 summarizes the docking analysis of lemongrass, clove, cassia and myrrh essential oils’ major compounds against scabies mite inactivated protease paralogue S-D1 (SMIPP-S-D1, PDB 3H7T). Myrrh essential oil components (β-ocimene, α-copaene, and *trans*-α-bisabolene) exhibited binding energies (ranging from − 5.5 to − 4.1 kcal/mol) and showed strong alkyl hydrophobic interactions with Leu 31, Leu 107, Lys 225, and Ile 228 amino acid residues (Fig. 5). Due to its hydroxyl moiety, α-santalol was the only one to have a hydrogen bond interaction with Lys 225 amino acid residue as the H-donor. In addition, it showed good binding affinity (− 4.9 kcal/mol) and displayed two alkyl hydrophobic interactions with Leu 107 and Lys 225 residues. As for the cassia essential oil components, both cinnamaldehyde derivatives showed the same interacting pattern with binding energies (ranging from − 4.2 to − 4.4 kcal/mol). They displayed one hydrogen bond with Lys 225 as the H-donor and two hydrophobic interactions (Leu 107 as (Pi-sigma) and Leu 31 (Pi-alkyl). In addition, the alkyne derivative showed the highest binding score (− 4.7 kcal/mol) among cassia essential oil components, with two hydrogen bonds with (Leu 31 and Ile 105) amino acids residues as H-acceptor and two hydrophobic interactions (Ile 228 (Pi-sigma) and Lys 225 (Pi-alkyl) (Fig. 4).

Eugenol attained hydrogen bond interaction with Lys 104 as an H-donor at the binding site. Moreover, eugenol, caryophyllene, and naphthalenone derivatives showed hydrophobic interactions with (Leu 107, Lys 225, and Ile 228) amino acid residues. The dehydrodieugenol B possessed the highest binding score (− 5.2 kcal/mol) with three hydrogen bond interactions (Lys 103, Gly 194, and Lys 104). It also showed hydrophobic interactions with Lys 11, Trp 14, and Leu 121. Similarly, the lemongrass essential oil components, geranial, neral, myrcene, and geraniol, all showed alkyl hydrophobic interactions, especially with (Leu 107, Lys 225, and Ile 228) amino acid residues. Remarkably, geraniol showed the highest binding energy − 4.5 kcal/mol and hydrogen bond interaction with Ile 105 as the H-acceptor.

The high binding affinity and in vitro biological activity might be owing to the similarity of (*E*)-cinnamaldehyde, cinnamaldehyde-dimethyl acetal, and 3-phenyl-2-propyn-1-ol from cassia with eugenol from clove flower bud. Their binding scores for both enzymes were comparable. Moreover, while β-ocimene (myrrh) and myrcene (lemongrass) differ only in the position of their respective double bonds, it is worth mentioning that β-ocimene has shown higher binding affinity for both enzymes. In addition, α-copaene (myrrh) which is structurally similar to caryophyllene (clove) has demonstrated a much stronger binding affinity for both enzymes. Collectively, essential oils from cassia bark and myrrh oleo gum resin will have an important role in the recovery of humans and animals from scabies mites.

## Conclusion

In conclusion, we conducted a study to determine the efficacy of three essential oils (cassia, myrrh, and fennel) as scabicides against *S. scabiei*. Additionally, we analyzed the primary active components of each essential oil using GC-MS. It was found that both cassia and myrrh essential oils have diverse components. The in vitro anti-scabies bioassay demonstrated both concentration- and time-dependent effects of cassia and myrrh essential oils with a significant anti-scabies activity even at 5% concentration. However, fennel essential oil did not show any lethal effect on *S. scabiei.* The docking analysis showed strong to moderate binding affinities of components from cassia and myrrh essential oils towards GST and SMIPP-S-D1 enzymes. Molecular dynamics simulation might be needed to further understand protein-ligand complex stability. Based on our findings, it can be inferred that cassia and myrrh essential oils offer effective and environmentally friendly alternatives to conventional scabies drugs, which can be harmful to both humans and animals. However, positive control drugs such as ivermectin should be included in further in vitro bioassay studies. In addition, further research is needed to identify the specific active ingredients and ensure the safety profile of these essential oils. Further studies on oxidative stress markers and inflammatory response of infected rabbits need to be performed. By addressing these aspects, we can better understand the efficacy and applicability of cassia and myrrh essential oils in scabies treatment.

## Electronic supplementary material

Below is the link to the electronic supplementary material.

Supplementary Material 1: Docking results of GSH, major constituents of lemongrass, clove flower bud, cassia bark, and myrrh essential oils into glutathione transferase, as well as, docking results of major constituents of lemongrass, clove flower bud, cassia bark, and myrrh essential oils into scabies mite inactivated protease paralogue S-D1

## Data Availability

The authors declare that the data supporting the findings of this study are available within the paper and its Supplementary Information files. Any raw data files are needed in another format they are available from the corresponding author upon reasonable request.

## References

[1] Nardoni S, Mancianti F. Essential Oils against Sarcoptes scabiei. Molecules. 2022;27(24). 10.3390/molecules27249067 PMC9788335 36558200

[2] Abbas A, Abbas R, Masood S, Iqbal Z, Khan M, Kashif M, Raza MA, Mahmood MS, Khan J. Sindhu ZuD: acaricidal and insecticidal effects of essential oils against ectoparasites of veterinary importance. Bol Latinoam Caribe Plant Med Aromat. 2018;17:441–52.

[3] Fang F, Candy K, Melloul E, Bernigaud C, Chai L, Darmon C, Durand R, Botterel F, Chosidow O, Izri A, et al. In vitro activity of ten essential oils against *Sarcoptes scabiei*. Parasites Vectors. 2016;9(1):594. 27876081 10.1186/s13071-016-1889-3 PMC5120413

[4] Walton SF, Holt DC, Currie BJ, Kemp DJ. Scabies: new future for a neglected disease. Advances in parasitology. Volume 57. ed.: Academic; 2004. pp. 309–76. 10.1016/S0065-308X(04)57005-7 15504541

[5] El-Khateeb EA, Imam AA, Sallam MA. Pattern of skin diseases in Cairo, Egypt. Int J Dermatol. 2011;50(7):844–53. 21699521 10.1111/j.1365-4632.2010.04840.x

[6] Hegab DS, Kato AM, Kabbash IA, Dabish GM. Scabies among primary schoolchildren in Egypt: a sociomedical environmental study in Kafr El-Sheikh administrative area. Clin Cosmet Investig Dermatol. 2015;8:105–11. 25759594 10.2147/CCID.S78287 PMC4345923

[7] Hegazy AA, Darwish NM, Abdel-Hamid IA, Hammad SM. Epidemiology and control of scabies in an Egyptian village. Int J Dermatol. 1999;38(4):291–5. 10321946 10.1046/j.1365-4362.1999.00630.x

[8] Andriantsoanirina V, Guillot J, Ratsimbason M, Mekhloufi G, Randriamialinoro F, Ranarivelo L, Ariey F, Durand R. In vitro efficacy of essential oils against *Sarcoptes scabiei*. Sci Rep. 2022;12(1):7176. 35504935 10.1038/s41598-022-11176-x PMC9065015

[9] Karimkhani C, Colombara DV, Drucker AM, Norton SA, Hay R, Engelman D, Steer A, Whitfeld M, Naghavi M, Dellavalle RP. The global burden of scabies: a cross-sectional analysis from the global burden of disease study 2015. Lancet Infect Dis. 2017;17(12):1247–54. 28941561 10.1016/S1473-3099(17)30483-8 PMC5700804

[10] Liuwan C, Listiawan M, Murtiastutik D, Ervianti E, Sawitri S, Prakoeswa C, Astari L, Ningrat F, Kurniati K, Fitriani W, et al. The effectiveness of 5% tea tree oil cream, 10% tea tree oil cream, and 5% permethrin cream for scabies treatment in pediatric patients. Berkala Ilmu Kesehatan Kulit Dan Kelamin. 2020;32:200.

[11] Pasay C, Mounsey K, Stevenson G, Davis R, Arlian L, Morgan M, Vyszenski-Moher D, Andrews K, McCarthy J. Acaricidal activity of Eugenol based compounds against scabies mites. PLoS ONE. 2010;5(8):e12079. 20711455 10.1371/journal.pone.0012079 PMC2920318

[12] Aboelhadid SM, Mahrous LN, Hashem SA, Abdel-Kafy EM, Miller RJ. In vitro and in vivo effect of *Citrus Limon* essential oil against sarcoptic mange in rabbits. Parasitol Res. 2016;115(8):3013–20. 27098160 10.1007/s00436-016-5056-8

[13] Oladimeji FA, Orafidiya OO, Ogunniyi TAB, Adewunmi TA. Pediculocidal and scabicidal properties of *Lippia multiflora* essential oil. J Ethnopharmacol. 2000;72(1):305–11. 10967487 10.1016/s0378-8741(00)00229-4

[14] Sharma DK, Saxena VK, Sanil NK, Singh N. Evaluation of oil of *Cedrus deodara* and benzyl benzoate in sarcoptic mange in sheep. Small Ruminant Res. 1997;26(1):81–5.

[15] Dimri U, Sharma MC. Effects of sarcoptic mange and its control with oil of *Cedrus deodara*, *Pongamia glabra, Jatropha curcas*, and benzyl benzoate, both with and without ascorbic acid on growing sheep: assessment of weight gain, liver function, nutrient digestibility, wool production and meat quality. J Vet Med Ser A. 2004;51(2):79–84. 10.1111/j.1439-0442.2004.00602.x 15153077

[16] Li M, Liu S, Yin Z, Bernigaud C, Guillot J, Fang F. Activity of terpenes derived from essential oils against *Sarcoptes scabiei* eggs. Parasites Vectors. 2021;14(1):600. 34886874 10.1186/s13071-021-05094-6 PMC8656058

[17] Batiha GE, Wasef L, Teibo JO, Shaheen HM, Zakariya AM, Akinfe OA, Teibo TKA, Al-Kuraishy HM, Al-Garbee AI, Alexiou A, et al. Commiphora Myrrh: a phytochemical and Pharmacological update. Naunyn Schmiedebergs Arch Pharmacol. 2023;396(3):405–20. 36399185 10.1007/s00210-022-02325-0 PMC9672555

[18] Massoud AM, El Ebiary FH, Abd El Salam NF. Effect of myrrh extract on the liver of normal and bilharzially infected mice. An ultrastructural study. J Egypt Soc Parasitol. 2004;34(1):1–21. 15125513

[19] Panthong S, Itharat A, Naknarin S, Kuropakornpong P, Ooraikul B, Sakpakdeejaroen I. Bactericidal Effect and Anti-Inflammatory Activity of *Cassia garettiana* Heartwood Extract. Scientific World Journal. 2020;2020:1653180. 10.1155/2020/1653180 PMC7374238 32765193

[20] Netopilova M, Houdkova M, Urbanova K, Rondevaldova J, van Damme P, Kokoska L. In vitro antimicrobial combinatory effect of *Cinnamomum Cassia* essential oil with 8-hydroxyquinoline against *Staphylococcus aureus* in liquid and vapor phase. J Appl Microbiol. 2020;129(4):906–15. 32350955 10.1111/jam.14683

[21] El Atki Y, Aouam I, El Kamari F, Taroq A, Nayme K, Timinouni M, Lyoussi B, Abdellaoui A. Antibacterial activity of cinnamon essential oils and their synergistic potential with antibiotics. J Adv Pharm Technol Res. 2019;10(2):63–7. 31041184 10.4103/japtr.JAPTR_366_18 PMC6474160

[22] Naaz S, Ahmad N, Qureshi MI, Hashmi N, Akhtar MS, MM AK. Antimicrobial and antioxidant activities of fennel oil. Bioinformation. 2022;18(9):795–800. 37426506 10.6026/97320630018795 PMC10326323

[23] Fahim SF. Effect of fennel oil on *Tetranychus urticae* (Acari: Tetranychidae) and two predatory mites. Int J Entomol. 2022;7(4):24–32.

[24] Liao F, Hu Y, Tan H, Wu L, Wang Y, Huang Y, Mo Q, Wei Y. Acaricidal activity of 9-oxo-10,11-dehydroageraphorone extracted from *Eupatorium adenophorum* in vitro. Exp Parasitol. 2014;140:8–11. 24631419 10.1016/j.exppara.2014.02.009

[25] McGrath JC, Drummond GB, McLachlan EM, Kilkenny C, Wainwright CL. Guidelines for reporting experiments involving animals: the ARRIVE guidelines. Br J Pharmacol. 2010;160(7):1573–6. 20649560 10.1111/j.1476-5381.2010.00873.x PMC2936829

[26] Mapara M, Thomas BS, Bhat KM. Rabbit as an animal model for experimental research. Dent Res J (Isfahan). 2012;9(1):111–8. 22363373 10.4103/1735-3327.92960 PMC3283968

[27] Li M, Liu B, Bernigaud C, Fischer K, Guillot J, Fang F. Lemongrass (*Cymbopogon citratus*) oil: A promising miticidal and ovicidal agent against *Sarcoptes scabiei*. PLoS Negl Trop Dis. 2020;14(4):e0008225. 32251453 10.1371/journal.pntd.0008225 PMC7162540

[28] Felgenhauer BE. Techniques for Preparing crustaceans for scanning electron microscopy. J Crustac Biol. 1987;7(1):71–6.

[29] Low WY, Feil SC, Ng HL, Gorman MA, Morton CJ, Pyke J, McConville MJ, Bieri M, Mok Y-F, Robin C, et al. Recognition and detoxification of the insecticide DDT by *Drosophila melanogaster* glutathione S-Transferase D1. J Mol Biol. 2010;399(3):358–66. 20417639 10.1016/j.jmb.2010.04.020

[30] Fischer K, Langendorf CG, Irving JA, Reynolds S, Willis C, Beckham S, Law RHP, Yang S, Bashtannyk-Puhalovich TA, McGowan S, et al. Structural mechanisms of inactivation in scabies mite Serine protease paralogues. J Mol Biol. 2009;390(4):635–45. 19427318 10.1016/j.jmb.2009.04.082

[31] Trott O, Olson AJ. AutoDock Vina: improving the speed and accuracy of Docking with a new scoring function, efficient optimization, and multithreading. J Comput Chem. 2010;31(2):455–61. 19499576 10.1002/jcc.21334 PMC3041641

[32] Sanner MF. Python: a programming Language for software integration and development. J Mol Graph Model. 1999;17(1):57–61. 10660911

[33] Meng EC, Goddard TD, Pettersen EF, Couch GS, Pearson ZJ, Morris JH, Ferrin TE. UCSF chimerax: tools for structure Building and analysis. Protein Sci. 2023;32(11):e4792. 37774136 10.1002/pro.4792 PMC10588335

[34] BIOVIA: Dassault Systèmes, Discovery studio visualizer,. In., vol. v24.1.0.23298. 2024 edn: San Diego; 2024.

[35] Bergström FC, Reynolds S, Johnstone M, Pike RN, Buckle AM, Kemp DJ, Fischer K, Blom AM. Scabies mite inactivated Serine protease paralogs inhibit the human complement system. J Immunol. 2009;182(12):7809–17. 19494305 10.4049/jimmunol.0804205

[36] Na YE, Kim SI, Bang HS, Kim BS, Ahn YJ. Fumigant toxicity of Cassia and cinnamon oils and cinnamaldehyde and structurally related compounds to *Dermanyssus gallinae* (Acari: Dermanyssidae). Vet Parasitol. 2011;178(3–4):324–9. 21324598 10.1016/j.vetpar.2011.01.034

[37] Kim HK, Yun YK, Ahn YJ. Fumigant toxicity of Cassia bark and Cassia and cinnamon oil compounds to *Dermatophagoides Farinae* and *Dermatophagoides Pteronyssinus* (Acari: Pyroglyphidae). Exp Appl Acarol. 2008;44(1):1–9. 18247142 10.1007/s10493-008-9129-y

[38] Nwanade CF, Wang M, Li H, Masoudi A, Yu Z, Liu J. Individual and synergistic toxicity of cinnamon essential oil constituents against *Haemaphysalis longicornis* (Acari: Ixodidae) and their potential effects on non-target organisms. Ind Crops Prod. 2022;178:114614.

[39] Massoud AM, Kutkat MA, Abdel Shafy S, El-Khateeb RM, Labib IM. Acaricidal efficacy of myrrh (*Commiphora molmol*) on the fowl tick Argas persicus (Acari: Argasidae). J Egypt Soc Parasitol. 2005;35(2):667–86. 16083075

[40] Elhalawany AS, Dewidar AA. Efficiency of Some Plant Essential Oils Against the Two-Spotted Spider Mite, *Tetranychus urticae* Koch and the Two Predatory Mites *Phytoseiulus persimilis* (A.-H.), and *Neoseiulus californicus* (McGregor). Egyptian Academic Journal of Biological Sciences A, Entomology. 2017;10(7):135–147.

[41] Burt S. Essential oils: their antibacterial properties and potential applications in foods-a review. Int J Food Microbiol. 2004;94(3):223–53. 15246235 10.1016/j.ijfoodmicro.2004.03.022

[42] Kiss T, Cadar D, Spînu M. Tick prevention at a crossroad: new and renewed solutions. Vet Parasitol. 2012;187(3–4):357–66. 22424918 10.1016/j.vetpar.2012.02.010

[43] Traina O, Cafarchia C, Capelli G, Iacobellis NS, Otranto D. In vitro acaricidal activity of four monoterpenes and solvents against *Otodectes Cynotis* (Acari: Psoroptidae). Exp Appl Acarol. 2005;37(1–2):141–6. 16180080 10.1007/s10493-005-0359-y

[44] Fang S. Insect glutathione S-transferase: a review of comparative genomic studies and response to xenobiotics. Bull Insectol. 2012;65:265–71.

[45] Reynolds SL, Fischer K. Pseudoproteases: mechanisms and function. Biochem J. 2015;468(1):17–24. 25940733 10.1042/BJ20141506

