# Supplementary material for: Investigating the potential scabicidal activities of three essential oils: gaining mechanistic insights through in vitro contact bioassay and molecular docking
Source: BMC Complement Med Ther. 2025 May 22;25:185. doi: 10.1186/s12906-025-04868-0 (PMC12100822; doi:10.1186/s12906-025-04868-0)
Supplement: Supplementary file 1 — Supplementary Material 1: Docking results of GSH, major constituents of lemongrass, clove flower bud, cassia bark, and myrrh essential oils into glutathione transferase, as well as, docking results of major constituents of lemongrass, clove flower bud, cassia bark, and myrrh essential oils into scabies mite inactivated protease paralogue S-D1 [file 12906_2025_4868_MOESM1_ESM.docx]

**Supporting information for**

Investigating the potential scabicidal activities of three essential oils: Gaining mechanistic insights through in vitro contact bioassay and molecular docking

Rofida Wahman^1,*^, Shaymaa Mohamed^1^, Soad A.L. Bayoumi^1^, Rana M. I. Morsy^2^, Salma E. Sayed^3^, Salma M. Darwish^3^, Nada S. Abdelraheem^3^, Fatma A. Mohamed^3^, Radwa Y. Ibrahim^4^, Norhan B. B. Mohammed^5^, Doaa A. Yones^4^, Sara Abdelal Mohammed^6^, and Alzahraa Abdelraouf Ahmad^4^

Affiliation

^1^Department of Pharmacognosy, Faculty of Pharmacy, Assiut University, Assiut, 71526, Egypt.

^2^Department of Pharmaceutical Organic Chemistry, Faculty of Pharmacy, Assiut University, Assiut, 71526, Egypt.

^3^Center for Pharmaceutical Studies and Research for Medicinal Plants, Faculty of Pharmacy student, Assiut University, Assiut, 71526, Egypt.

^4^Department of Parasitology, Faculty of Medicine, Assiut University, Assiut, 71515, Egypt.

^5^Department of Medical Biochemistry, Faculty of Medicine, South Valley University, Qena, 83523 Egypt.

^6^Department of Veterinary Parasitology, Faculty of Veterinary Medicine, Assiut University, Assiut, 71515, Egypt.

Correspondence

***Rofida Wahman,** rofidawahman@aun.edu.eg

Contents:

Table S1 …………………………………………………………………………………………………… 2

Table S2 …………………………………………………………………………………………………….4

**Table S1**. Docking results of GSH, major constituents of lemongrass, clove flower bud, cassia bark, and myrrh essential oils into S. scabiei glutathione transferase represented by Drosophila melanogaster Delta class GST. PDB code: 3EIN.

| Compound name | *score S*^a^ (kcal/mol) | Hydrogen bond interaction | Distance  (Å) | Hydrophobic interactions | Distance (Å) |
| --- | --- | --- | --- | --- | --- |
| GSH  (glutathione) | -5.4 | - The 52/H-donor  - Ile 53/  (two H-donor)  - Glu 65/H-donor  - Arg 67/  (two H-donor) | 2.50  2.72 and  2.21  2.20  2.23 and  2.84 | --------- | --------- |
| lemongrass | | | | | |
| Geranial | -6.0 | - Ser 11/H-Donor  - Tyr 106/H-Donor | 2.05  2.67 | - Leu 7/Alkyl  - Pro 8/Alkyl  - Tyr 207/Pi-Alkyl  - Phe 204/Pi-Alkyl | 3.73  4.90  5.32  5.32 |
| Neral | -5.6 | - Ser 10/H-Donor | 3.02 | - Phe 118;  Pi-Sigma  Pi-Alkyl  - Tyr 207/Pi-Alkyl | 3.78  4.71  4.66 |
| Myrcene | -5.8 | --------- | --------- | - Leu 7  (two Alkyl)  - Pro 8/Alkyl  - Phe 118  (two Pi-Alkyl)  - Phe 204  (two Pi-Alkyl)  - Tyr 207  (three Pi-Alkyl) | 4.99 and  3.72  4.47  5.01 and  4.79  5.07and  5.12  4.92,  4.36 and  5.47 |
| Geraniol | -5.9 | - Glu 203 / H-acceptor | 2.96 | - Leu 7/Alkyl  - Tyr 114/Pi-Alkyl  - Phe 118/Pi-Alkyl  - Phe 204/Pi-Alkyl  - Tyr 207/Pi-Alkyl | 5.39  5.17  5.04  4.88  5.05 |
| clove flower bud | | | | | |
| Eugenol | -5.9 | - Tyr 114/H-Donor  - Ser 10/Carbon H-Acceptor | 2.46  3.53 | - Tyr 114/Pi- H.B  - Phe 118/Pi-Pi Stacked  - Pro 12/Alkyl  - Tyr 207/Pi-Alkyl | 3.35  5.58  4.47  4.99 |
| Caryophyllene | -7.4 | --------- | --------- | - Tyr 207/Pi-Sigma  - Leu 7/Alkyl | 3.93  3.89 |
| Dehydrodieugenol B | -7.3 | - Tyr 114/H-Donor  - Ser 10/ Carbon H-Acceptor | 2.45  3.64 | - Pro 12/Alkyl  - Leu 34/  Alkyl  Pi-Alkyl  - His 39/Pi-Alkyl  - Tyr 207  (two Pi-Alkyl) | 4.55  4.30  5.36  4.57  4.88 and  5.03 |
| 7-Isopropyl-4*α*-methyloctahydro-2(1H)-naphthalenone | -7.7 | - Ser 11/H-Donor  - Tyr 114/H-Donor  - Pro 12/Carbon H-Donor | 2.84  2.46  3.52 | --------- | --------- |
| cassia bark | | | | | |
| (*E*)-Cinnamaldehyde | -5.8 | - Ser 10/ Carbon H-donor | 3.53 | - Tyr 207/  Pi-Pi T-shaped  - Leu 7/Pi-Alkyl | 4.78  5.10 |
| Cinnamaldehyde-dimethyl acetal | -6.0 | - Ser 10/ Carbon H-donor | 3.67 | - Tyr 207/  Pi-Pi T-shaped  - Leu 7/Pi-Alkyl | 4.89  5.14 |
| 3-Phenyl-2-propyn-1-ol | -6.1 | - Ser 10 / Carbon H-donor | 3.34 | - Gly 9/  Pi-Pi T-shaped  - Leu 7/Pi-Alkyl | 4.31  5.15 |
| Benzaldehyde | -5.3 | - Ser 11/H-donor  - Tyr 106/H-donor  - Tyr 114/H-donor | 2.73  2.71  2.30 | - Phe 204/  Pi-Pi T-shaped | 4.90 |
| Myrrh | | | | | |
| *β*-Ocimene | -6.0 | --------- | --------- | - Leu 7  (two Alkyl)  - Pro 8/Alkyl  - Tyr 114/Pi-Alkyl  - Phe 118/Pi-Alkyl  - Phe 204  (two Pi-Alkyl)  - Tyr 207/Pi-Alkyl | 4.89 and  3.64  4.15  4.89  4.99  5.06 and  5.09  5.42 |
| *α*-Copaene | -7.7 | --------- | --------- | - Leu 7  (three alkyl)  - Leu 34/Alkyl  - Phe 118/pi-Alkyl  - Tyr 207  (two pi-Alkyl) | 5.17,  4.83 and  3.70  5.06  4.44  4.77 and  4.56 |
| *trans* -*α*-Bisabolene | -7.7 | --------- | --------- | - Leu 7/Alkyl  - Pro 12/Alkyl  - Phe 204/pi-Alkyl  - Tyr 207/pi-Alkyl | 5.02  4.38  5.27  4.98 |
| *α*-Santalol | -6.6 | - Tyr 106/H-acceptor  - Ser 10/ Carbon H-acceptor | 3.03  3.48 | - Tyr 207/  Pi-Sigma  Pi-Alkyl  - Leu 7/Alkyl | 3.94  5.21  5.04 |

^a^S: The score of a compound placement inside the protein binding pocket.

**Table S2**. Docking results of major constituents of lemongrass, clove flower bud, cassia bark, and myrrh essential oils into scabies mite inactivated protease paralogue S-D1 (SMIPP-S-D1) PDB code: 3H7T.

| Compound name | *score S*^a^ (kcal/mol) | Hydrogen bond interaction | Distance  (Å) | Hydrophobic interactions | Distance (Å) |
| --- | --- | --- | --- | --- | --- |
| lemongrass | | | | | |
| Geranial | -4.3 | - Ser 229/H-donor | 2.66 | - Leu 107/ (two Alkyl)  - Lys 225/ Alkyl  - Ile 228/ Alkyl  - Leu 31/ Alkyl | 5.16 and  3.80  4.87  4.17  3.99 |
| Neral | -4.0 | - Leu 107/H-donor  - Glu 106/H-donor | 2.00  2.60 | - Leu 107/Alkyl  - Lys 225/ (two Alkyl)  - Ile 228/ (two Alkyl) | 5.22  5.26 and 4.22  4.40 and  4.29 |
| Myrcene | -3.6 | --------- | --------- | - Leu 31/  (two Alkyl)  - Leu 107/  (two Alkyl)  - Ile 228/  (two Alkyl)  - Lys 225/ Alkyl | 5.49 and  3.99  4.88 and  3.74  4.61 and  4.89  4.00 |
| Geraniol | -4.5 | - Ile 105/H-acceptor | 2.35 | - Leu 107/ Alkyl  - Ile 228/ (two Alkyl)  - Lys 225/ Alkyl | 4.82  5.19 and  4.52  4.37 |
| clove flower bud | | | | | |
| Eugenol | -4.5 | - Lys 104/H-donor | 3.09 | - Lys 225/ Alkyl  - Ile 228/ Alkyl  - Leu 107/ Pi-Alkyl | 3.78  3.90  5.25 |
| Caryophyllene | -4.8 | --------- | --------- | - Leu 107/ Alkyl  - Lys 225/ Alkyl | 5.00  5.48 |
| Dehydrodieugenol B | -5.2 | - Lys 103/H-Donor  - Gly 194/ Carbon H-Donor  - Lys 104/ Carbon H-Acceptor | 2.50  3.67  3.78 | - Trp 14/  Pi-Pi T-shaped  Pi-Alkyl  - Leu 121/ Alkyl  - Lys 11/ Alkyl  - Lys 103/ Alkyl | 4.72  4.86  4.52  4.35  4.13 |
| 7-Isopropyl-4*α*-methyloctahydro-2(1H)-naphthalenone | -4.9 | - Leu 107/H-Donor | 1.85 | - Ile 228/  Alkyl | 5.04 |
| cassia bark | | | | | |
| (*E*)-Cinnamaldehyde | -4.2 | - Lys 225/H-Donor | 2.04 | - Leu 107/  Pi-Sigma  - Leu 31/ Pi-Alkyl | 3.56  5.36 |
| Cinnamaldehyde-dimethyl acetal | -4.4 | - Lys 225/H-Donor | 2.20 | - Leu 107/  Pi-Sigma  - Leu 31/ Pi-Alkyl | 3.59  5.39 |
| 3-Phenyl-2-propyn-1-ol | -4.7 | - Leu 31/H- acceptor  - Ile 105/H- acceptor | \| 2.88 \| \| --- \| \| 2.40 \| | - Ile 228/ Pi-Sigma  - Lys 225/ Pi-Alkyl | 3.71  5.07 |
| Benzaldehyde | -4.0 | - Asp 99/H-Donor | 2.22 | - Leu 97/  Pi-Alkyl  - Lys 103/ Pi-Alkyl  - Lys 104/ Pi-Alkyl | 4.52  4.35  5.06 |
| Myrrh | | | | | |
| *β*-Ocimene | -4.1 | --------- | --------- | - Lys 225/ (two Alkyl)  - Ile 228/ (two Alkyl)  - Leu 107 (two Alkyl)  - Leu 31/Alkyl | 4.87 and  4.17  4.00 and  4.56  4.37 and  3.71  3.95 |
| *α*-Copaene | -5.5 | --------- | --------- | - Leu 107/ (two Alkyl)  - Ile 228/ (two Alkyl)  - Leu 31/ Alkyl  - Lys 225/ Alkyl | 4.67 and 3.72  4.91 and 4.00  3.85  3.93 |
| *trans* -*α*-Bisabolene | -5.2 | --------- | --------- | - Leu 107/ (two Alkyl)  - Lys 225/ (two Alkyl)  - Ile 228/ Alkyl  - Leu 31/ Alkyl | 4.53 and  4.06  3.87 and 4.31  5.40  4.54 |
| *α*-Santalol | -4.9 | - Lys 225/H- acceptor | 2.65 | - Leu 107/ Alkyl  - Lys 225/ Alkyl | 5.21  3.70 |

^a^S: The score of a compound placement inside the protein binding pocket.
